# Structural Studies and Thermal Analysis in the Cs_2_MoO_4_–PbMoO_4_ System with Elucidation of β-Cs_2_Pb(MoO_4_)_2_

**DOI:** 10.1021/acs.inorgchem.3c00241

**Published:** 2023-04-25

**Authors:** Andries van Hattem, John Vlieland, Robert Dankelman, Michel A. Thijs, Gilles Wallez, Kathy Dardenne, Jörg Rothe, Rudy J.M. Konings, Anna L. Smith

**Affiliations:** † Radiation Science and Technology Department, Faculty of Applied Sciences, Delft University of Technology, Mekelweg 15, Delft 2629JB, The Netherlands; ‡ Sorbonne University, Pierre and Marie Curie Campus, Paris 06, Paris 75005, France; ¶ Institute for Nuclear Waste Disposal (INE), Radionuclide Speciation Department, Karlsruhe Institute of Technology (KIT), Hermann-von-Helmholtz-Platz 1, Eggenstein-Leopoldshafen 76344, Germany

## Abstract

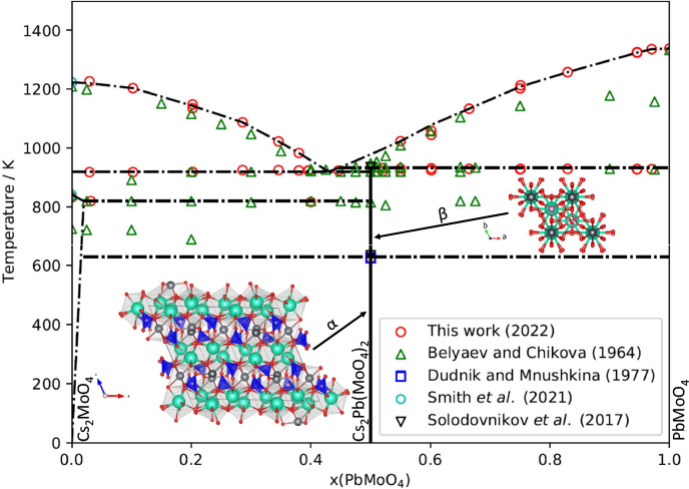

The quaternary compound Cs_2_Pb(MoO_4_)_2_ was synthesized and its structure was characterized using X-ray and neutron diffraction from 298 to 773 K, while thermal expansion was studied from 298 to 723 K. The crystal structure of the high-temperature phase β-Cs_2_Pb(MoO_4_)_2_ was elucidated, and it was found to crystallize in the space group *R*3̅*m* (No. 166), i.e., with a palmierite structure. In addition, the oxidation state of Mo in the low-temperature phase α-Cs_2_Pb(MoO_4_)_2_ was studied using X-ray absorption near-edge structure spectroscopy. Phase diagram equilibrium measurements in the Cs_2_MoO_4_–PbMoO_4_ system were performed, revisiting a previously reported phase diagram. The equilibrium phase diagram proposed here includes a different composition of the intermediate compound in this system. The obtained data can serve as relevant information for thermodynamic modeling in view of the safety assessment of next-generation lead-cooled fast reactors.

## Abstract 2

The Cs_2_MoO_4_−PbMoO_4_ phase diagram, including the Cs_2_Pb(MoO_4_)_2_ intermediate, was investigated. Phase equilibria were obtained using differential scanning calorimetry. α-Cs_2_Pb(MoO_4_)_2_ was investigated using X-ray absorption near-edge structure spectroscopy and high-temperature X-ray diffraction, while the structure of β-Cs_2_Pb(MoO_4_)_2_ was elucidated for the first time using neutron and X-ray diffraction.

## Introduction

The structural family of binary molybdates and tungstates shows appealing properties, for instance, ferroelastic and ferroelectric behavior.^1,2^ Among these complex compounds is a group of binary molybdates and tungstates with mono- and bivalent elements, i.e., the A_2_^+^B^2+^(X^6+^O_4_)_2_ structural type, with A = K, Rb, or Cs, B = Ba and Pb, and X = Mo and W.^1,3−8^ Moreover, binary molybdates and tungstates are also versatile as host materials for phosphors.^9,10^

Our research into Cs_2_Pb(MoO_4_)_2_ is motivated by the need for safe, clean, and affordable energy, which is a prerequisite for sustainable societies. The lead-cooled fast reactor (LFR), one of the six nuclear reactor designs selected by the Generation IV International Forum,^11^ has the potential for such energy production. The first operative class of lead-cooled reactors was used in the former USSR for submarine propulsion. However, these submarines were prematurely decommissioned because of corrosion issues.^12^ Since then, research has continued in other countries too, e.g., with the MYRRHA accelerator driven system in Belgium, the design of which is based on cooling with a lead–bismuth eutectic.^13,14^ The currently envisioned designs for the next-generation LFRs are cooled with either liquid lead (Pb) or a eutectic mixture of lead and bismuth, while the reference fuel in Europe is a mixed oxide [(U,Pu)O_2_] fuel^15^ with Pu content ranging between 15 and 30%.^16^ Features of the LFR include a fast-neutron spectrum and operation with a closed fuel cycle, allowing for actinide recycling.^11^

Because postirradiation studies of the LFR are not known, the fission product chemistry is assumed to be similar to that in sodium-cooled fast reactors (SFRs) with comparable linear heating rates. For SFRs, experience was gained within, e.g., the Phenix project.^17^ During irradiation, numerous fission products are generated within the fuel matrix, including gases, metallic precipitates, oxide precipitates, and fission products soluble inside the fuel matrix.^16,18^ Of particular interest in this research is the class of volatile and semivolatile elements (Cs, Mo, I, and Te) that migrate from the center of the (U,Pu)O_2_ fuel pellet toward the fuel periphery because of the expected very high temperatures and thermal gradient (over 2273 K in the center and 873 K in the pellet rim with a gradient of over 1000 K·cm^–1^).^16^ Those fission products accumulate with time in the space between fuel and cladding and form above 7–8 atom % burn-up a so-called Joint Oxide Gaine (JOG) layer of a few hundred micrometers, with mostly Cs_2_MoO_4_ in combination with CsI and Cs_2_Te according to postirradiation examinations and thermochemical calculations.^17,19,20^

Before the LFR can be built, a comprehensive accident scenario analysis needs to be performed. One of the possible accidental scenarios is the breach of the cladding material during reactor operation, in which case the coolant will come into contact with the irradiated fuel, starting with the JOG layer for a burn-up higher than 7–8 atom %. Therefore, one of the possible chemical interactions that needs investigation for the safety assessment is the interaction of Pb coolant with Cs_2_MoO_4_, a major JOG-phase constituent. With this scenario in mind, the chemistry of the Pb–Cs–Mo–O system is investigated. In this study, the emphasis is on the pseudobinary section Cs_2_MoO_4_–PbMoO_4_ of the ternary system PbO–Cs_2_O–MoO_3_, which includes the quaternary phase Cs_2_Pb(MoO_4_)_2_.

**Figure 1:**
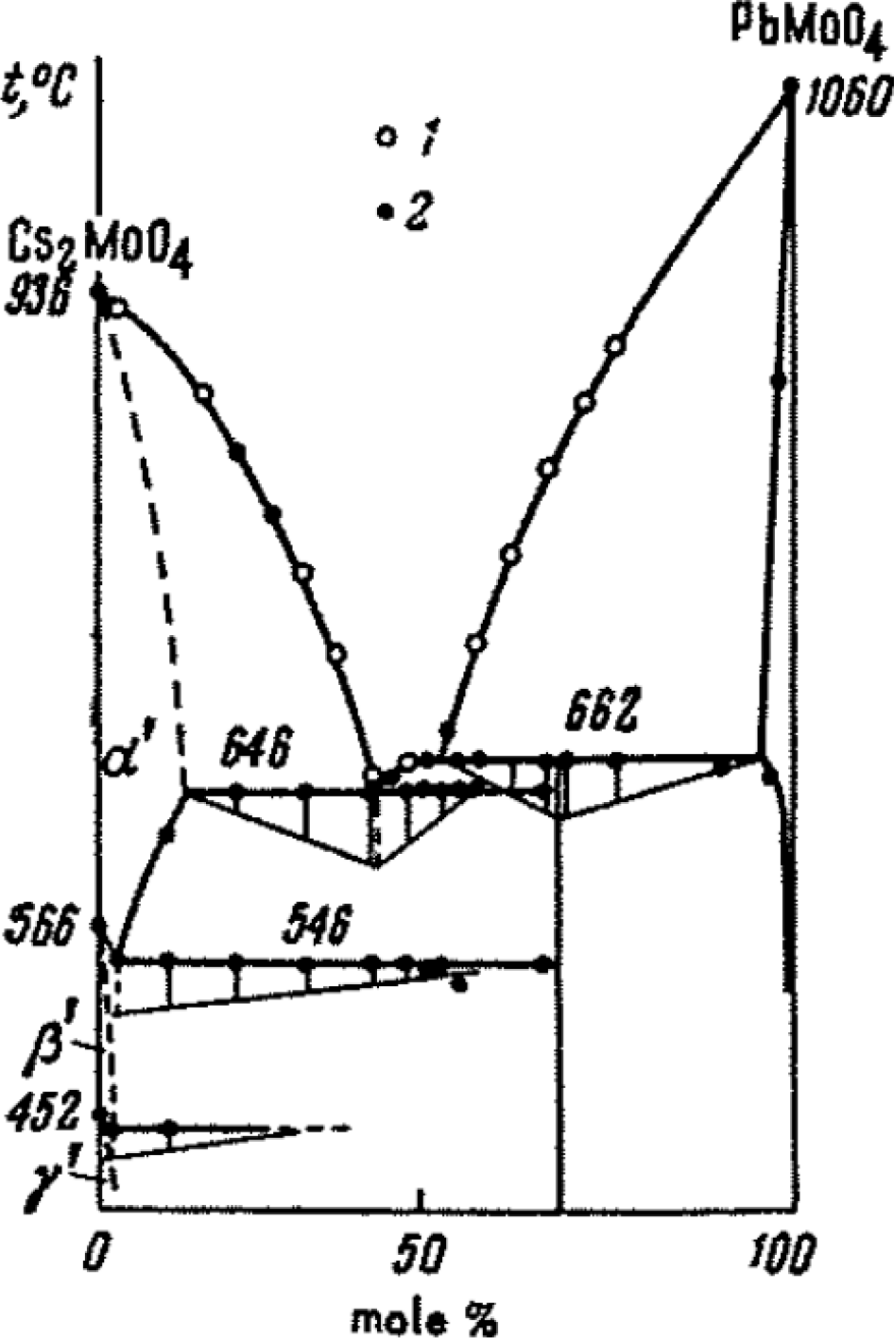
Cs_2_MoO_4_–PbMoO_4_ phase diagram as reported by Belyaev and Chikova.^21^ The open circles correspond to visual polythermal results; the closed circles correspond to thermographic results. Reproduced from ref (21). Copyright 1964 Springer.

The literature on the phase diagram Cs_2_MoO_4_–PbMoO_4_ is, to the best of our knowledge, limited to a study by Belyaev and Chikova published in 1964.^21^ Their published phase diagram is reproduced in Figure 1. Their interpretation of the measured phase equilibria can be related to their experimental approach, but their claim of the existence of Cs_2_Pb_2_(MoO_4_)_3_, which they report to decompose at 935 K, is found to be incorrect. They also suggest solid solubility domains near the end members, which we will discuss in light of our new investigations.

In 1977, Dudnik and Mnushkina investigated K_2_Pb(MoO_4_)_2_ and isomorphous compounds, among which is Cs_2_Pb(MoO_4_)_2_.^3^ They grew crystals in this series without studying complete crystallographic details. They made the specific composition and, using polarized light, determined that a domain structure disappeared above a certain temperature. Based on this, they reported a phase transition at 626 ± 10 K for Cs_2_Pb(MoO_4_)_2_. The compound was also mentioned in a review paper on binary molybdates by Solodovnikov et al. in 1994.^4^ In a later review on molybdates and tungstates of monovalent and bivalent elements by Isupov,^1^ both transition temperatures are mentioned, i.e., that at 626 ± 10 K (phase transition) and that at 935 K (decomposition) for the Cs_2_Pb(MoO_4_)_2_ stoichiometry. The compound Cs_2_Pb(MoO_4_)_2_ was also found in a study by Tsyrenova et al. in 1987.^22^ The first dedicated crystallographic study on Cs_2_Pb(MoO_4_)_2_ was published by Solodovnikov et al. in 2017, based on single-crystal data.^7^ The authors elucidated the crystal structure and performed differential scanning calorimetry (DSC) to study the thermal behavior. Moreover, they investigated the electronic properties of the compound, which is out of the scope of the current research.

This work brings new insights to the phase equilibria in the Cs_2_MoO_4_–PbMoO_4_ system, solves discrepancies noticed in the literature, and explores in more detail the structural and thermal properties of the quaternary phase Cs_2_Pb(MoO_4_)_2_, using X-ray and neutron diffraction (XRD and ND, respectively), DSC, and X-ray absorption spectroscopy (XAS). Thereby, it positions the compound Cs_2_Pb(MoO_4_)_2_ in the updated phase diagram section Cs_2_MoO_4_–PbMoO_4_ and explores the properties of relevance of Cs_2_Pb(MoO_4_)_2_ for a LFR safety assessment.

## Experimental Section

### Synthesis

Cs_2_MoO_4_ was synthesized from Cs_2_CO_3_ (99.99%, Alfa Aesar) and MoO_3_ (99.5%, Alfa Aesar) by means of a solid-state reaction. The precursors were mixed stoichiometrically, ground thoroughly, and heated twice for 12 h at 973 K in an alumina crucible under an oxygen atmosphere. The sample was reground intermittently. PbMoO_4_ (99.9%) was purchased from Merck Sigma. Cs_2_Pb(MoO_4_)_2_ was synthesized by mixing Cs_2_MoO_4_ and PbMoO_4_ in a 1:1 ratio. After thorough grinding, the mixture was heated in an alumina crucible for 12 h at 773 K. After cooling, the mixture was reground and heated for 12 h at 873 K. Several batches were prepared; in general, the synthesis was performed under an oxygen atmosphere; one synthesis was done under an argon flow. The purity was estimated to be higher than 99%; only some faint peaks (*I*/*I*_max_ < 0.1%) were found. The presence of other intermediate compounds in the Cs_2_MoO_4_–PbMoO_4_ system was investigated by synthesis attempts as used for Cs_2_Pb(MoO_4_)_2_ at various molar fractions *x*(PbMoO_4_) between 0.0 and 0.5 and between 0.5 and 1.0. Typically, the samples were heated to 773 and 873 K for 12 h each.

### XRD

The purity of the synthesized end members and Cs_2_Pb(MoO_4_)_2_ was confirmed by powder XRD using a PANalytical X’Pert PRO X-ray diffractometer mounted in the Bragg–Brentano configuration with a copper anode (0.4 mm × 12 mm line focus, 45 kV, 40 mA). The data were collected using an X’celerator detector in the angle range 10° ≤ 2θ ≤ 120° with a 0.008° step size in 2θ. The total measurement time was about 7 h. The samples were loaded in airtight sample holders closed with Kapton foil to prevent powder spreading of the toxic Pb compound and to avoid reaction with moisture. Structural analysis was performed on the diffraction patterns using the profile refinement method^23,24^ in the *FullProf* suite.^25^ For Cs_2_Pb(MoO_4_)_2_ refinements, the parameters as given by Solodovnikov et al.^7^ were taken as a starting point.

High-temperature (ht-)XRD was done using the same XRD instrument equipped with an Anton Paar TTK450 sample holder. The sample chamber was evacuated. The sample was measured at room temperature and from 323 K to 723 K with an increment of 50 K. The total measurement time was about 4 h per set temperature.

### ND

ND was performed on Cs_2_Pb(MoO_4_)_2_ at the PEARL beamline^26^ at the Hoger Onderwijs Reactor at the Delft University of Technology. The sample was encapsulated in a vanadium null–alloy container hermetically closed with a rubber O-ring. The data were collected at room temperature and 573 and 773 K with a fixed wavelength of 0.166718 nm in the angle range 11° ≤ 2θ ≤ 159°. Data analysis was performed using the profile refinement method^23,24^ in the *FullProf* suite.^25^

### X-ray Absorption Near-Edge Structure (XANES) Spectroscopy

XANES spectroscopy measurements were performed for Cs_2_MoO_4_, PbMoO_4_, and Cs_2_Pb(MoO_4_)_2_ at the INE-Beamline^27^ of KIT Light Source (Karlsruhe, Germany) with 2.5 GeV and 150–170 mA as operating conditions in the Karlsruhe Research Accelerator (KARA) storage ring. The beamline uses a Ge(422) double-crystal monochromator (DCM). Rh-coated mirrors before (flat, cylindrically bent) and after (toroidal) the DCM are used to collimate and focus the synchrotron beam, respectively, producing a spot size of 500 μm × 500 μm at the sample surface. Transmission and fluorescence geometries could be measured in unison. Samples were probed around the K-edge of Mo (20 keV). XAS samples were prepared by mixing some of the compound with boron nitride (BN), which around the Mo K-edge is almost transparent to X-rays. The samples mixed with BN were pressed into a circular pellet of 8 mm diameter and enclosed in Kapton foil.

The energy *E*_0_ of the edge absorption threshold position was taken at the inflection point of the spectrum using the zero crossing of the second derivative. The position of the prepeak was selected as the peak maximum, using the zero crossing of the first derivative. Several acquisitions were performed on the same sample and summed up to improve the signal-to-noise ratio. Before the scans were averaged, each spectrum was aligned using the XANES spectrum of a metallic Mo reference foil measured simultaneously. *ATHENA* software^28^ was used to normalize and analyze the spectra.

### DSC

Phase diagram measurements in the Cs_2_MoO_4_–PbMoO_4_ system were performed using simultaneous thermogravimetry (TG) analysis with DSC using the TG–DSC module of a Setaram Multi HTC 96-line calorimeter with plate-type sensors. In general, (mixtures of) the compounds Cs_2_Pb(MoO_4_)_2_, Cs_2_MoO_4_, and PbMoO_4_ were measured with a sample size of about 100 mg of powder in an open alumina cup under an oxygen atmosphere. The heating ramp used was 10 K·min^–1^. The temperature on the heating ramp was calibrated and corrected for the effect of the heating rate by measuring the melting points of standard high-purity metals (In, Sn, Pb, Al, Ag, and Au) at 2, 4, 5, 8, 10, and 12 K·min^–1^. The transition temperatures were derived on the heating ramp as the onset temperature using tangential analysis of the recorded heat flow if the event was interpreted as polymorphism, congruent melting, or eutectic, while liquidus event temperatures were based on the peak maximum. The uncertainty on the measured temperatures was estimated to be ±5 K for pure compounds and ±10 K for mixtures.

The solid solubility near the end members was studied by mixing Cs_2_MoO_4_ or PbMoO_4_ with Cs_2_Pb(MoO_4_)_2_ to mole fractions *x*(PbMoO_4_) = 0.03 and *x* = 0.97, respectively. At *x*(PbMoO_4_) = 0.03, the mixture was heated until 1023 K with 10 K·min^–1^, stabilized at that temperature for 30 min, cooled to 773 K, and three times heated and cooled between 773 and 1023 K with stabilization of 30 min after each heating and cooling. At *x*(PbMoO_4_) = 0.97, a similar procedure was carried out, with 823 and 1023 K as the set temperatures. In this way, the samples were cycled around the phase transitions in the region of interest without crossing the liquidus line.

The melting point of PbMoO_4_ was also measured using a Setaram Multi-Detector HTC Module of the 96-line calorimeter with 3D heat flux detection. Open alumina cups were used under an oxygen flow around ambient pressure. The temperature on the heating ramp was calibrated using the same procedure as that for TG–DSC. The melting point was based on determination of the onset temperature of the event. The estimated uncertainty was ±5 K.

## Results and Discussion

### Structural Characterization of α-Cs_2_Pb(MoO_4_)_2_ by ND and XRD

**Figure 2:**
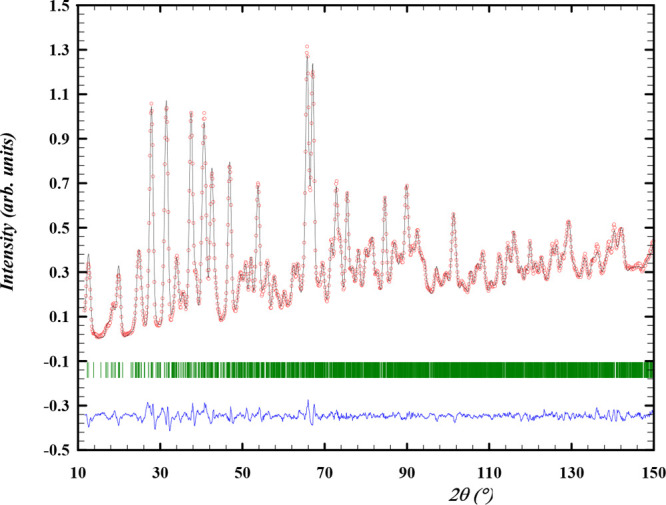
Experimental (*Y*_obs_, in red) and calculated (*Y*_calc_, in black) ND patterns of α-Cs_2_Pb(MoO_4_)_2_ at room temperature. The difference between the calculated and experimental intensities *Y*_obs_ – *Y*_calc_ is shown in blue. The angular positions of the Bragg reflections are shown in green. Measurement at λ = 1.66718 Å.

**Figure 3:**
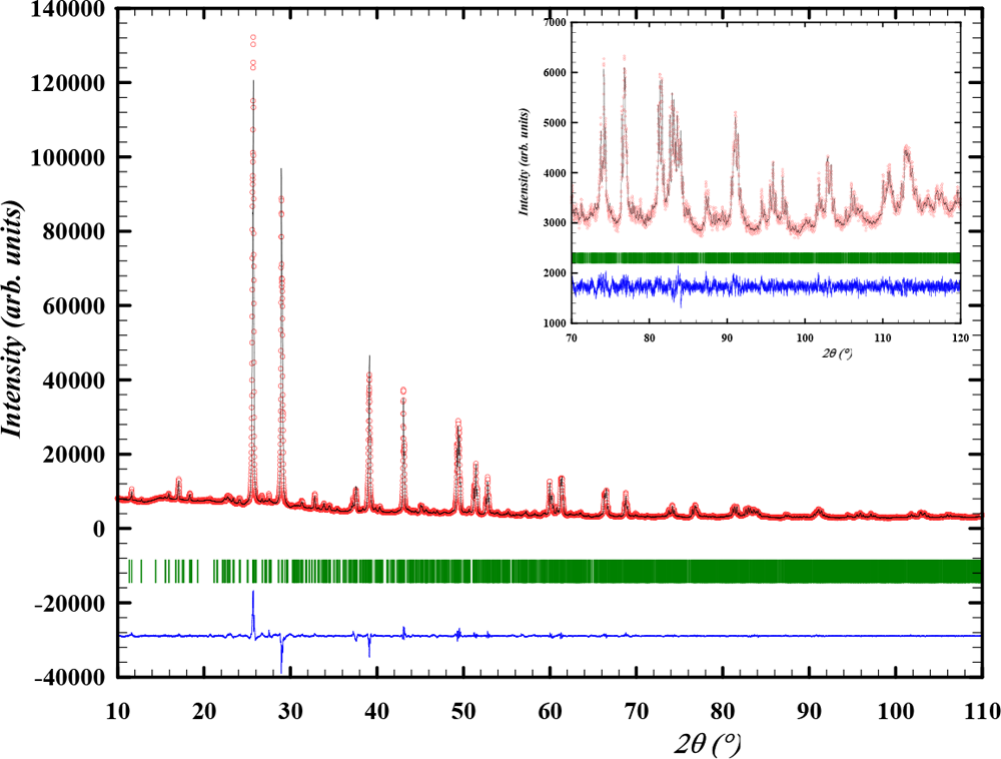
Experimental (*Y*_obs_, in red) and calculated (*Y*_calc_, in black) XRD patterns of α-Cs_2_Pb(MoO_4_)_2_ at 298 K. The difference between the calculated and experimental intensities *Y*_obs_ – *Y*_calc_ is shown in blue. The angular positions of the Bragg reflections are shown in green. Measurement at λ = Cu K_α_.

**Figure 4:**
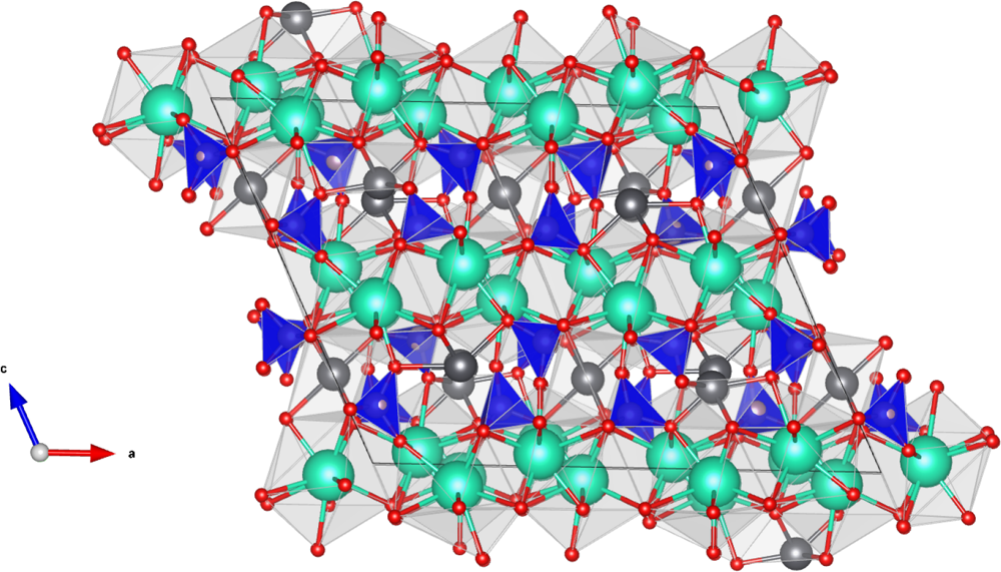
Room-temperature crystal structure of α-Cs_2_Pb(MoO_4_)_2_ as refined in this work from ND data. The MoO_4_^2–^ tetrahedra are in blue, the Pb atoms are in gray, the Cs atoms are in green, and the O atoms are in red. The visualization was made using *Vesta*.^29^

The diffraction patterns obtained with X-rays and neutrons are shown in Figures 2 and 3, respectively, while the refined cell parameters obtained with both methods are listed in Table 1. α-Cs_2_Pb(MoO_4_)_2_ crystallizes with monoclinic symmetry in the space group *C*2/*m* (No. 12); the atomic coordinates as reported by Solodovnikov et al. were used as a starting point for the refinement.^7^ The ND data were refined here by imposing soft constraints on the Mo–O distances (fixed at 1.79 Å ± 0.02%) and atomic displacement parameters (one *B* value per chemical element). The values obtained for the atomic displacement parameters are high but rather realistic considering that Cs-based structures are soft and subject to thermal displacement. A refinement without any constraints was first attempted but would lead to unphysical Mo–O bond lengths (scattering between 1.41 and 2.07 Å) considering the covalency of the bond. The quality of the refinement is considered satisfactory given that it is performed on powders for a large unit cell with low symmetry, meaning that there are many atomic positions (35 in total) and substantial peaks overlap. The crystal structure (Figure 4) can be described as consisting of two types of layers: layers of corner-sharing Cs polyhedra and layers of alternating Pb polyhedra and molybdate tetrahedra that are corner-sharing. The coordination number for Cs cations is 10; the Pb cations have an irregular 6-fold coordination.

**Table 1:** Cell Parameters of α-Cs_2_Pb(MoO_4_)_2_ as Determined in This Research with XRD and ND and as Reported in the Literature^7^ as Measured at Ambient Conditions^a^

synthesis atmosphere	method	*a*^b^/Å	*b*^b^/Å	*c*^b^/Å	β^b^/deg	*V*/Å^3^
O_2_	XRD	21.3860(19)	12.2784(11)	16.7944(16)	114.994(5)	3997.3(6)
Ar	XRD	21.3752(23)	12.2879(13)	16.7916(19)	114.998(8)	3997.3(8)
O_2_	ND	21.345(14)	12.257(7)	16.760(10)	114.98(6)	3975(4)
not reported	XRD^7^	21.3755(13)	12.3123(8)	16.8024(10)	115.037 (2)	4006.6(3)

a The compound crystallizes in the monoclinic space group *C*2/*m* (No. 12).

b Note that the statistically derived standard uncertainties obtained from the refinement were underestimated by about 1 order of magnitude and were thus multiplied by 10, as listed in this table.

The XRD pattern was refined using the atomic positions found from the refined neutron pattern. It is observed that the fitted cell parameters for ND are slightly smaller than those obtained by XRD. The same effect was observed in the XRD and ND refined profile parameters of Cs_2_Ba(MoO_4_)_2_ by Smith et al.^8^ The discrepancy, which is about the same on the three axes, might be explained by a slight deviation in the neutron wavelength or from a refinement artifact. A temperature difference during the measurement cannot account for the order of magnitude of this observation. The volume at 298 K that we found here from the XRD data deviated only 0.23% from the one reported by Solodovnikov et al.^7^ The choice of the synthesis gas (oxygen vs argon) seems to have no significant effect on the unit cell parameters. With these diffraction studies at 298 K, the recent results of Solodovnikov et al.^4^ with regard to the composition of the compound in the phase diagram Cs_2_MoO_4_–PbMoO_4_ are confirmed and further substantiated. It is interesting to note that Isupov^2^ silently corrected the composition of Belyaev and Chikova^21^ and/or used the one reported by Dudnik and Mnushkina^3^ because the author did no experimental investigations by that time and Dudnik and Mnushkina did not comment on the composition given by Belyaev and Chikova.

**Figure 5:**
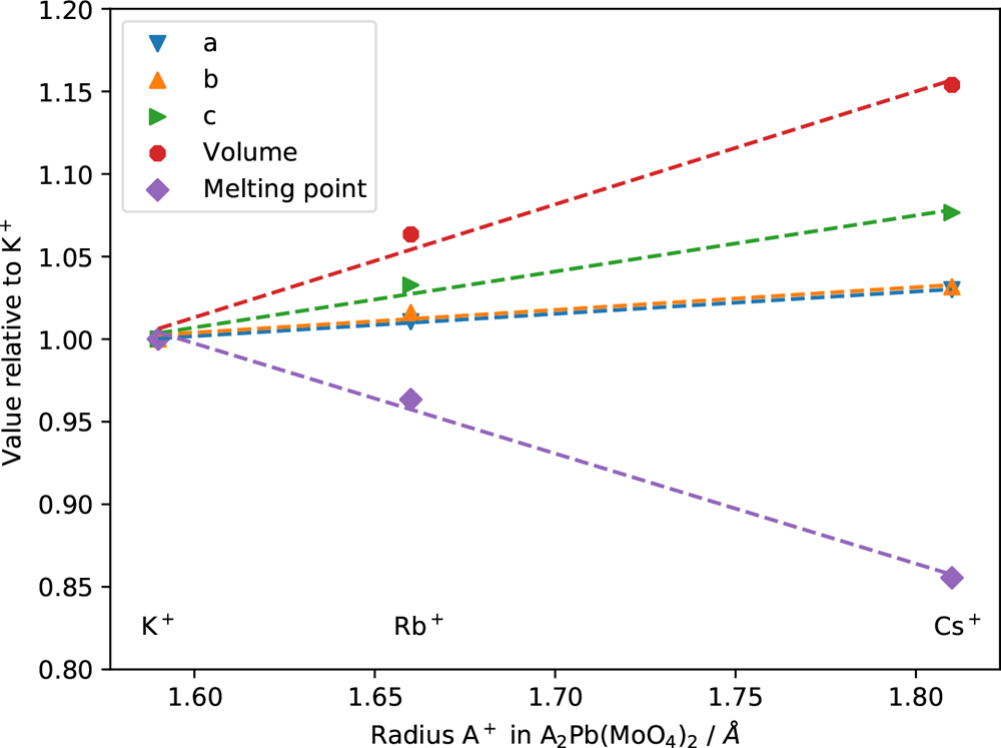
Trends of the cell parameters *a*, *b*, and *c*, volume, and melting point against the ionic radius of the alkali-metal ion with coordination number 10 for A_2_Pb(MoO_4_)_2_ (A = K, Rb, and Cs) using the crystal data from refs (5), (6), and (21) and the ionic radii from ref (30). The K^+^ values are used as the normalization factor.

The recent literature on A_2_Pb(MO_4_)_2_ with A = Cs, Rb, and K states that all three compounds crystallize in a large palmieriete-related superstructure.^5,6^ Unfortunately, the atomic positions for K_2_Pb(MoO_4_)_2_ have not been reported, so neither are the specific average alkali metal–oxygen distance in the coordination in the actual compound. However, using the ionic radii of the alkali-metal ion with a specific coordination number taken from the Shannon database,^30^ the unit cell parameters can be plotted against the ionic radius, as is done in Figure 5. For the Cs variant, the Cs coordination number is reported to be 10^7^, while for Rb, the coordination number is equal to 10–12. For K, we do not have the data. The cell parameters increase approximately linearly, while the melting point decreases with increasing ionic radius of the alkali-metal ion.^5,6,21^

### Structural Characterization of β-Cs_2_Pb(MoO_4_)_2_ by ND and XRD

**Figure 6:**
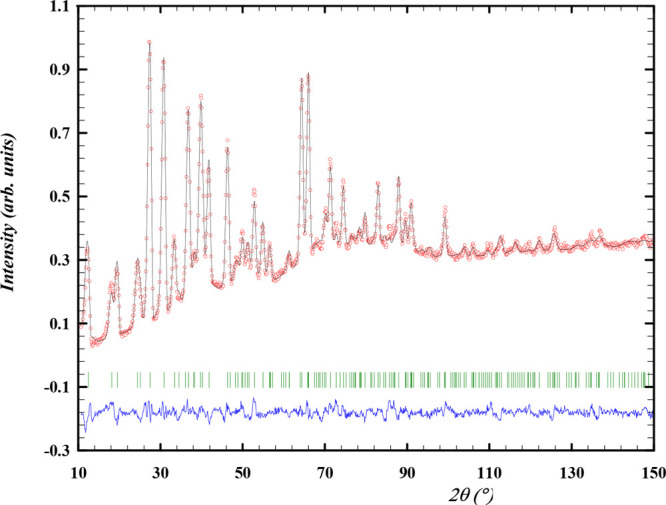
Experimental (*Y*_obs_, in red) and calculated (*Y*_calc_, in black) ND patterns of β-Cs_2_Pb(MoO_4_)_2_ at 773 K. The difference between the calculated and experimental intensities *Y*_obs_ – *Y*_calc_ is shown in blue. The angular positions of the Bragg reflections are shown in green. Measurement at λ = 1.66718 Å.

**Figure 7:**
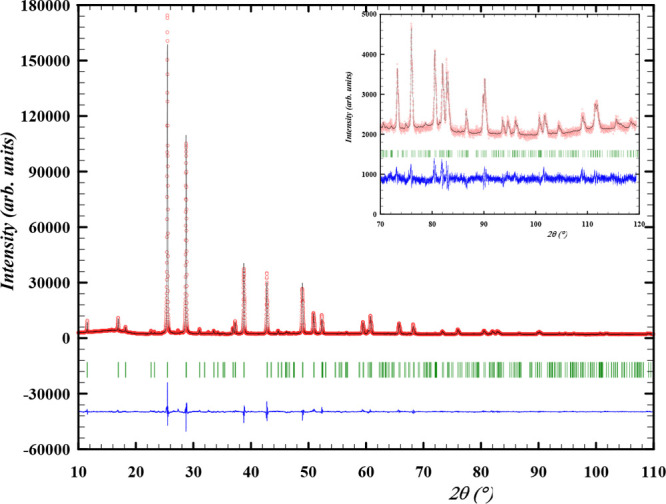
Experimental (*Y*_obs_, in red) and calculated (*Y*_calc_, in black) XRD patterns of β-Cs_2_Pb(MoO_4_)_2_ at 723 K. The difference between the calculated and experimental intensities *Y*_obs_ – *Y*_calc_ is shown in blue. The angular positions of the Bragg reflections are shown in green. Measurement at λ = Cu K_α_.

**Figure 8:**
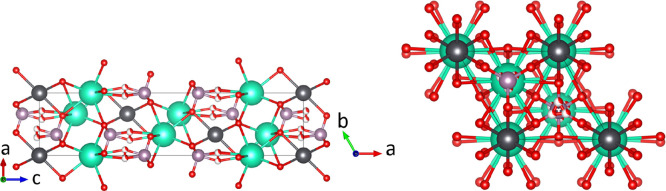
Crystal structure of β-Cs_2_Pb(MoO_4_)_2_ as refined in this work from the ND data at 773 K. The Pb atoms are in gray, the Cs atoms are in green, the Mo atoms are in purple, and the O atoms are in red. Partially white O atoms correspond to partial occupancy. Left: Visualization along the *b* axis. Right: Visualization along the *c* axis. The visualization was made using *Vesta*.^29^

The diffraction patterns obtained with neutrons at 773 K and X-rays at 723 K are shown in Figures 6 and 7, respectively, while the refined cell parameters obtained by both methods are listed in Table 2. For the refinement at high temperature, soft constraints on the Mo–O distances still had to be applied; the atomic displacements, however, are high, thus lowering the reliability of the intensities at high angles. Anisotropic displacement parameters were refined for each atom; the rather high values are justified by the high temperature, combined with the weakness of the Cs–O bonds. The position of the O1 atom splits into three positions around the axial site due to thermal disorder. The atomic coordinates, based on ND at 773 K, are given in Table 3, along with the occupancy factors and atomic displacement parameters. Visualizations along the *b* and *c* axes of β-Cs_2_Pb(MoO_4_)_2_ are given in Figure 8. In the diffraction patterns obtained above, the transition temperature and the reflections with at least one odd Miller index (*h*, *k*, or *l* = 2*n* + 1 with *n* a positive integer) disappear. This disappearance of the superstructure led us to refine β-Cs_2_Pb(MoO_4_)_2_ in the same space group [viz., *C*2/*m* (No. 12)] with halved cell parameters. In this way, the 2 and *m* symmetries are preserved as expected for a second-order phase transition; their number is even multiplied due to the shortening of the cell parameters. The *C* mode still applies, as is evident from the systematic extinctions. However, this new unit cell of β-Cs_2_Pb(MoO_4_)_2_ actually has a higher symmetry element: it crystallizes in the space group *R*3̅*m* (No. 166). Thus, the transition from α-Cs_2_Pb(MoO_4_)_2_ to β-Cs_2_Pb(MoO_4_)_2_ is the transition of the palmierite-related room-temperature phase to the actual palmierite structure at high temperature. In the Fourier difference map, some residuals were found that are believed not to correspond to the mother structure of β-Cs_2_Pb(MoO_4_)_2_. These residuals at a few percent level, which are ascribed to defects, together with the thermal displacement of the atoms, are reflected in the average quality of the refinements. A dedicated high-resolution single-crystal study would be desirable, but it is out of scope for the current research.

**Table 2:** Cell Parameters of β-Cs_2_Pb(MoO_4_)_2_ as Determined in This Research with XRD at 673 and 723 K and ND at 773 K^a^

*T*/K	method	*a*^b^/Å	*c*^b^/Å	*V*/Å^3^
673	XRD	6.2057(8)	22.968(3)	766.0(2)
723	XRD	6.2126(8)	22.977(3)	768.02(17)
773	ND	6.26(4)	23.00(25)	781(11)

a The compound crystallizes in the space group *R*3̅/*m* (No. 166). Here, *a* = *b* and γ = 120°.

b Note that the statistically derived standard uncertainties obtained from the refinement were underestimated by about 1 order of magnitude and were thus multiplied by 10, as listed in this table.

**Table 3:** Refined Atomic Positions in β-Cs_2_Pb(MoO_4_)_2_ Derived from the ND Pattern Refinement at 773 K^a^

atom	Wyckoff	*x*	*y*	*z*	occupancy factor	*B*_eq_/Å^2^
Pb	3a	0	0	0	1	6.3(4)
Cs	6c	0	0	0.198(4)	1	3(4)
Mo	6c	0	0	0.398(4)	1	4(4)
O1	18h	0.10(2)	0.05(2)	0.324(6)	0.3333	10(10)
O2	18h	0.302(6)	0.151(3)	0.4212(18)	1	9(2)

a *B*_eq_ = 8π^2^*U*_eq_, with *U*_eq_ refined by *FullProf*.

### Thermal Expansion of Cs_2_Pb(MoO_4_)_2_

**Figure 9:**
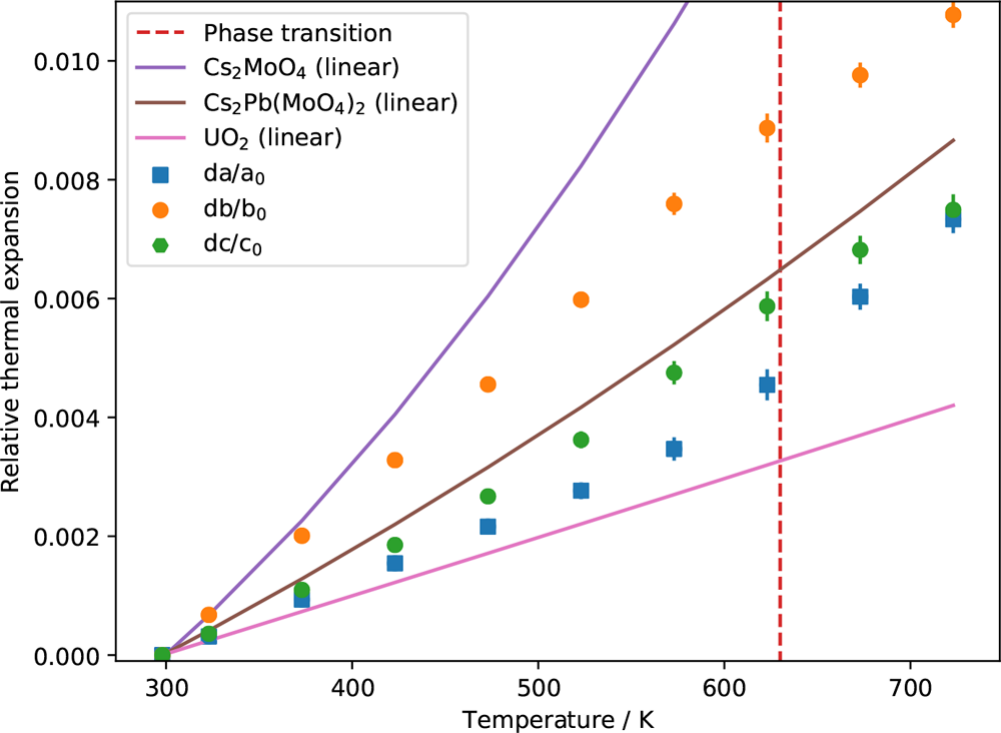
Relative thermal expansion of Cs_2_Pb(MoO_4_)_2_ based on XRD at various temperatures. The vertical dashed line is the second-order transition temperature, calculated as the average of refs (3) and (7), i.e., 630 K. If not visible, the error bars are smaller than the symbols. The relative figures are calculated using *f*(*T*) = (*x*_*T*_ – *x*_298 K_)/*x*_298 K_ with *x* = {*a*, *b*, *c*}.

For nuclear reactor applications, the thermal expansion behavior is of paramount significance to assess mechanical interaction of the possible product of a JOG–coolant interaction with the nuclear fuel and cladding. The refined cell parameters of Cs_2_Pb(MoO_4_)_2_ as evolving with temperature are given in Table 4. As can be concluded from this table, Cs_2_Pb(MoO_4_)_2_ shows a positive thermal expansion in the measured temperature range. The expansion is high due to the weakness of the Cs–O bonds, although the overall expansion is not as high as that in Cs_2_MoO_4_ because of the lower Cs content (for comparison, see Figure 9). The relative thermal expansion as calculated using *f*(*T*) = (*x*_*T*_ – *x*_298 K_)/*x*_298 K_ with *x* = {*a*, *b*, *c*}, is shown in Figure 9 for the whole temperature range 298–723 K, refining the XRD data in the space group *C*2/*m* (No. 12). A correction was applied for the fact that the high-temperature structure has halved cell parameters, and it contains only ^1^/_8_ of the atoms of the room-temperature structure. Linear relative expansion coefficients of the axes for *a*, *b* and *c* for the temperature range from 298 to 723 K are 16 × 10^–6^, 26 × 10^–6^, and 18 × 10^–6^ K^–1^, respectively. The mean relative thermal expansion d*l*/*l*_0_, taking *l*_0_ = *V*_0_^1/3^ at room temperature as the reference is given by
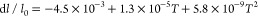
1for the temperature range from 298 to 723 K. The volumetric thermal expansion is defined as . When the phase transition is neglected, the volumetric thermal expansion is found to be 58.4 × 10^–6^ K^–1^ over the range 298–723 K. Besides ht-XRD, the ND data collected at 573 and 773 K also showed positive thermal expansion coefficients. The refined cell parameters at 298, 573, and 773 K are given in Tables 2 and 4. The ND data are available from the CCDC as 2239227 and 2239228 for the structures at respectively 298 and 773 K.

**Table 4:** Refined Cell Parameters and Unit Cell Volume for α-Cs_2_Pb(MoO_4_)_2_ as Measured by ht-XRD and ht-ND

*T*/K	*a*^a^/Å	*b*^a^/Å	*c*^a^/Å	β^a^/deg	*V*/Å^3^
XRD Data					
298	21.3860(18)	12.2791(10)	16.7929(15)	114.990(5)	3997.0(6)
323	21.3928(18)	12.2874(11)	16.7989(15)	114.991(5)	4002.4(6)
373	21.4060(19)	12.3038(11)	16.8113(16)	114.993(5)	4013.1(6)
423	21.4191(21)	12.3194(13)	16.8240(17)	114.998(6)	4023.5(7)
473	21.4323(23)	12.3351(14)	16.8377(19)	115.007(8)	4034.1(8)
523	21.445(3)	12.3526(17)	16.8537(24)	115.023(10)	4045.6(10)
573	21.460(4)	12.3724(23)	16.873(3)	115.045(18)	4058.7(14)
623	21.483(6)	12.388(3)	16.891(4)	115.072(26)	4071.9(18)
ND Data					
298	21.345(14)	12.257(7)	16.760(10)	114.98(6)	3975(4)
573	21.4(2)	12.39(13)	16.86(12)	115.1(8)	4053(70)

a Note that the statistically derived standard uncertainties obtained from the refinement were underestimated by about 1 order of magnitude and were thus multiplied by 10, as listed in this table.

As reported earlier, the phase transition in Cs_2_Pb(MoO_4_)_2_ is of second-order: based on the ferroelastic response, the transition temperature is reported to be 626 ± 10 K;^3^ based on DSC, the temperature is reported to be 635 ± 2 K;^7^ based on crystal optical observations on a polarizing microscope, it is reported to be 640 ± 2 K.^7^ In the ht-XRD measurements, a change was observed in the diffraction pattern by a change of the relative intensity in specific Bragg reflections *vide supra*. The high-temperature diffraction studies confirm the phase transition, as up to 623 K; the diffraction pattern can best be explained by α-Cs_2_Pb(MoO_4_)_2_, while the patterns at higher temperature are best explained by β-Cs_2_Pb(MoO_4_)_2_, as judged from the χ^2^ values. Close inspection of the evolution of the cell parameters seems to hint at a change of the linear response around 573 K. When the cell parameter *a* is plotted against temperature (Figure 9), the points from 298 K up to 523 K show an almost perfect linear increase; the correlation coefficient decreases slightly when the higher temperature points are included. The mean linear expansion evolves continuously at the transition point, which corresponds with the classification of a second-order phenomenon.

The mean relative thermal expansion is compared with Cs_2_MoO_4_ as a model for the JOG phase and UO_2_ as model for the fuel. For Cs_2_MoO_4_, the data were taken from Wallez et al.^31^ The recommended value for the thermal expansion of UO_2_^32,33^ is plotted in Figure 9. For Pu contents up to 30%, the thermal expansion of UO_2_ is representative of the mixed oxide fuel thermal expansion.^32^ As can be concluded from the plotted lines, the thermal expansion of Cs_2_Pb(MoO_4_)_2_ is approximately half of the expansion of the JOG phase but about twice as high as the fuel expansion. Thus, a potential formation of this quaternary phase in accidental conditions should not aggravate the mechanical interaction with the cladding compared to Cs_2_MoO_4_.

### XAS

In a LFR, the formation of Cs_2_Pb(MoO_4_)_2_ depends on the oxygen chemical potential of the fuel and the amount of oxygen dissolved in the Pb coolant. The oxidation state of Mo is key to this understanding and can be studied using XANES spectroscopy.

**Figure 10:**
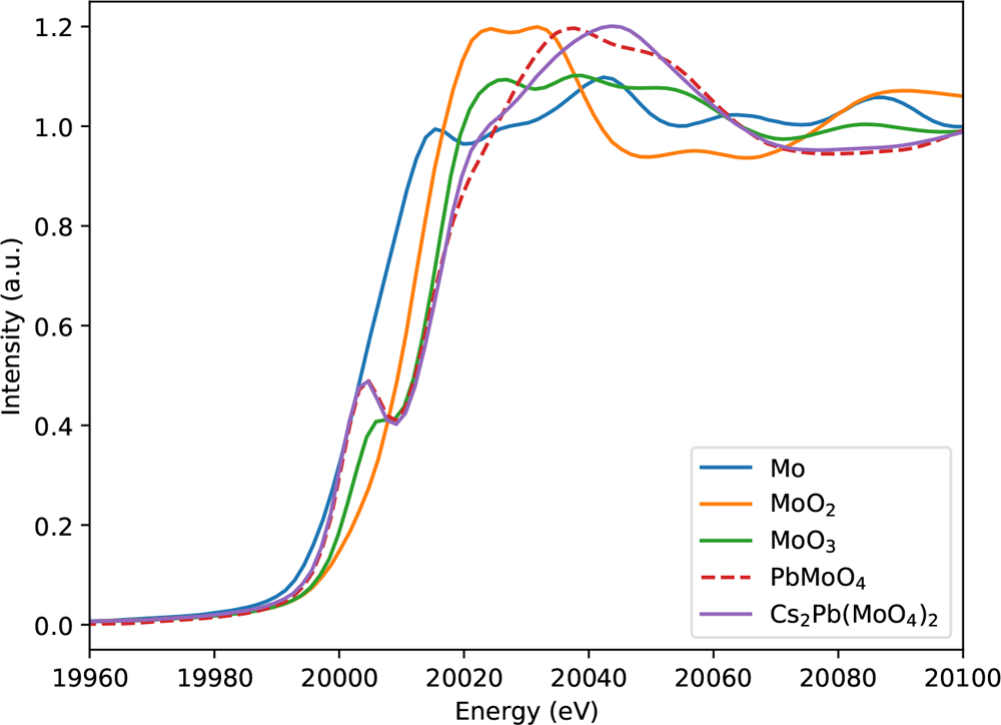
XANES spectra around the Mo K-edge.

In Figure 10, the collected XANES spectra around the Mo K-edge are shown. The derived absorption edge threshold and prepeak features are listed in Table 5. The intrinsic features of Mo(0), Mo(IV) in MoO_2_, and Mo(VI) in MoO_3_ (reference materials) can be seen in the increase in the *E*_0_ position with increasing Mo valence state. Moreover, while the former two have a simple edge, Mo(VI) in MoO_3_ has a characteristic preedge feature, which is explained below.

**Table 5:** Energies of the Prepeak and Edge in the Normalized XANES Spectra^a^

compound	prepeak (eV)	edge *E*_0_ (eV)
Mo(0)		20000
MoO_2_		20012.3
MoO_3_	20007.6	20015.8
PbMoO_4_	20004.5	20014.4
Cs_2_Pb(MoO_4_)_2_	20004.2	20016.4
MoO_2_^36^		20012.0
MoO_3_^36^	20007.6	20015.7
BaMoO_4_^36^	20006.1	20015.1

a The edges are determined based on the inflection points in the normalised XANES spectra at the Mo K-edge. The prepeaks are determined via the maximum. The estimated expanded uncertainty (with a coverage factor *k* = 2) on the energies is 1.0 eV.

The absorption at the K-edge involves a transition originating from the 1s orbital. Mo metal has the electronic configuration Kr 5s^1^4d^5^5p^0^. The transitions 1s → 4d and 1s → 5s are parity-forbidden. The transition of 1s → 5p is parity-allowed because when using the dipole approximation for the interaction of the X-rays with one electron,  is nonzero.

If the center of inversion on Mo is lost by distortion of an octahedron or switching to tetrahedral symmetry, hybridization of 4d with 5p causes a preedge to appear.^34^ In the molybdates, this extra edge feature is more pronounced than that in MoO_3_. In MoO_3_, this is observed as a shoulder due to core–hole broadening; the broadening comes from the hybridization of O 2p with Mo 4d and Mo 5p.^35^ The measured *E*_0_ values (Table 5) for PbMoO_4_ and Cs_2_Pb(MoO_4_)_2_ are very close to that of MoO_3_, indicating a valence state of 6+.

### Phase Equilibria in the Cs_2_MoO_4_–PbMoO_4_ Phase Diagram

#### Transition Temperatures of the End Members and Intermediate Compound Cs_2_Pb(MoO_4_)_2_

The transition temperatures measured in this work for the end-member compounds Cs_2_MoO_4_ and PbMoO_4_ and intermediate compound Cs_2_Pb(MoO_4_)_2_ are tabulated in Table 6 and compared to the literature.

**Table 6:** Phase Transition Temperatures of the Compounds in the Pseudobinary Section Cs_2_MoO_4_–PbMoO_4_ Measured in This Research Compared to Those in the Literature^a^

*x*(PbMoO_4_)	compound	*T*/K	transition type	method	ref
0.0	Cs_2_MoO_4_	837 ± 10	polymorphism	TG–DSC	this work
		839	polymorphism	TG	(21)
		841.3	polymorphism	CALPHAD model	(37)
		839 ± 5	polymorphism	TG–DSC	(38)
		1222 ± 5	congruent melting	TG–DSC	this work
		1209		TG	(21)
		1223	congruent melting	CALPHAD model	(37)
		1225 ± 5	congruent melting	TG–DSC	(38)
0.5	Cs_2_Pb(MoO_4_)_2_	626 ± 5	polymorphism	TG–DSC	this work
		626 ± 10	polymorphism	optical	(3)
		635 ± 2	polymorphism	DSC	(7)
		640 ± 2	polymorphism	optical	(7)
		923	melting	DTA	(22)
		933 ± 5	incongruent melting	TG–DSC	this work
		935	incongruent melting	TG	(21)
		926 ± 3	incongruent melting	DSC	(7)
1.0	PbMoO_4_	1340 ± 5	congruent melting	TG–DSC	this work
		1338 ± 5	congruent melting	DSC	this work
		1338	congruent melting		(39)
		1336	congruent melting	TG	(40)
		1343	congruent melting	DTA	(41)
		1336 ± 5	congruent melting	TG	(42)

a TG–DSC = thermogravic differential scanning calorimetry; TG = thermogravimetry; DTA = differential thermal analysis; optical = optical microscopy.

Cs_2_MoO_4_ was investigated many times, and a thermodynamic assessment in combination with a CALPHAD model was published by Smith et al. in 2021.^37^ The temperatures corresponding to the phase transition α-Cs_2_MoO_4_ → β-Cs_2_MoO_4_ and the melting points found during the current investigations (837 ± 5 and 1222 ± 5 K) match the values in the Cs–Mo–O CALPHAD model (841.3 and 1223 K) well. For comparison, the experimentally measured values of Belyaev and Chikova^21^ and Smith et al. are also given in Table 6.^38^

The melting point of PbMoO_4_ as measured in this work (1340 ± 5 and 1338 ± 5 K measured by TG–DSC and DSC) is in good agreement with the literature data.^39−42^ No polymorphism was observed for PbMoO_4_.

**Figure 11:**
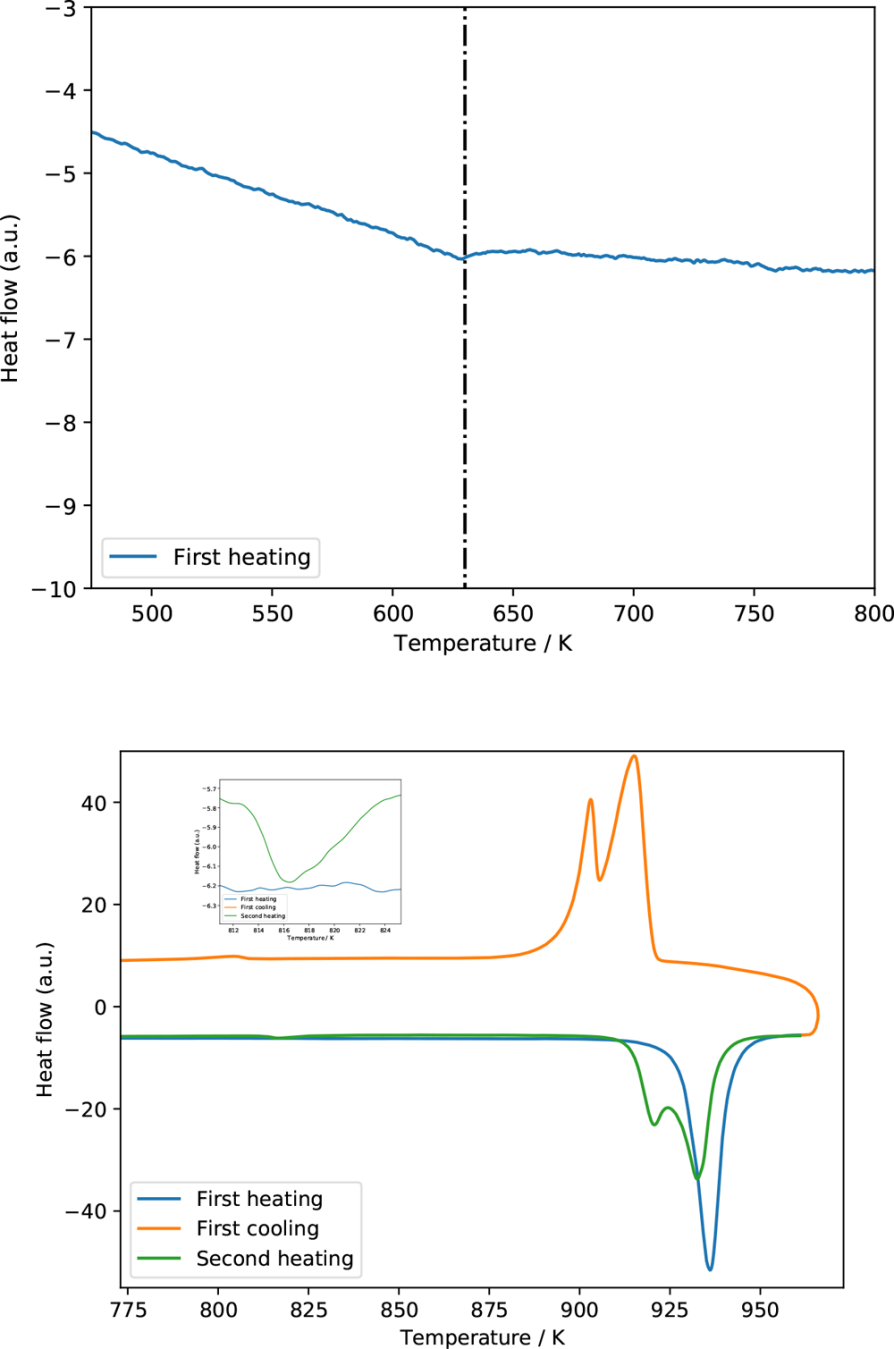
DSC curves of Cs_2_Pb(MoO_4_)_2_. Left: Temperature region in which a polymorphic transition of Cs_2_Pb(MoO_4_)_2_ is reported. The vertical dashed line is drawn at 630 K. Right: Temperature region of the polymorphic transition of Cs_2_MoO_4_, together with the decomposition peak of Cs_2_Pb(MoO_4_)_2_. The inset shows the presence of a solid solution of Pb in Cs_2_MoO_4_ formed after the first heating. For an explanation, see the text.

For Cs_2_Pb(MoO_4_)_2_, only a single event was detected upon heating, viz., incongruent melting of the compound. The second-order transition that was detected using DSC by Solodovnikov et al. is only present as a very small feature (Figure 11) in the current measurement with a 100 mg sample. Given the nature of the transition, no significant heat effect is expected. As a result, DSC is not the most appropriate technique for studying this transition. The derived temperature for the transition is 626 ± 5 K. Upon heating above the temperature of the large event in the DSC curve of Cs_2_Pb(MoO_4_)_2_, decomposition starts. This is evident from the cooling curve (also added in Figure 11), which shows two peaks close together and the appearance of a third peak. During the next heating cycle, it turns out that the temperature of the lower transition appears close to the polymorphism of Cs_2_MoO_4_ (it is actually somewhat lowered because of the solid solution, *vide infra*), while the splitting of the main peak is interpreted as the eutectic signal toward the Cs_2_MoO_4_-rich side and the peritectic toward the PbMoO_4_-rich side.

The presence of other compounds was excluded by the synthesis attempts; every time, a mixture of Cs_2_Pb(MoO_4_)_2_ with either Cs_2_MoO_4_ or PbMoO_4_ was found by XRD. Therefore, the number of compounds in this section is limited to the end members and the 1:1 compound.

#### Solid Solubility

Following the notation of Belyaev and Chikova for a moment^21^ (Figure 1), the solid solubility of PbMoO_4_ in Cs_2_MoO_4_ is 13% in the high-temperature phase (α′), 1.5% in the medium-temperature phase (β′), and 0.5% in the room-temperature phase (γ′). Their distinction between the medium- and room-temperature phase is not supported in the more recent literature,^1,31^ so γ′ can be neglected. Recent literature describes the room-temperature phase as α-Cs_2_MoO_4_, while the single high-temperature phase is denoted as β-Cs_2_MoO_4_.^37^ As described in the Experimental Section, the solubility of PbMoO_4_ in Cs_2_MoO_4_ was tested at mole fraction *x*(PbMoO_4_) = 0.03 and the solubility of Cs_2_MoO_4_ in PbMoO_4_ at mole fraction *x*(PbMoO_4_) = 0.97.

**Figure 12:**
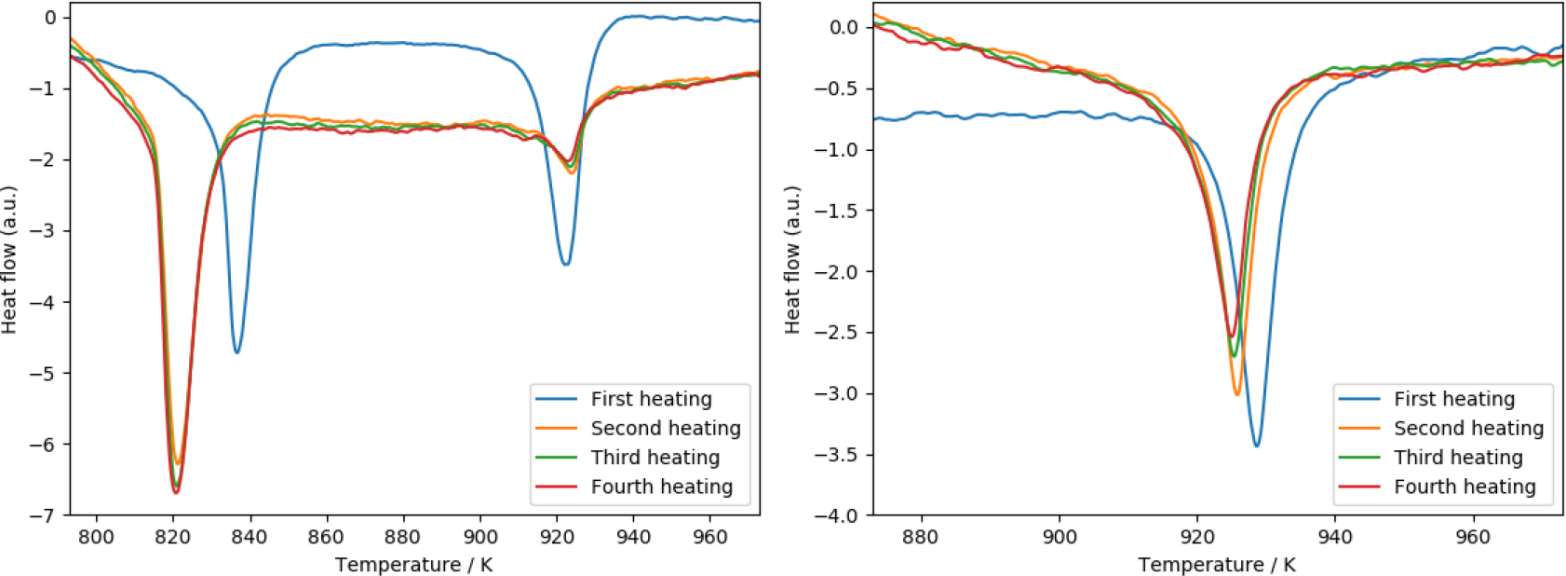
Heat-flow curves of mixtures of Cs_2_MoO_4_ or PbMoO_4_ with a slight amount of Cs_2_Pb(MoO_4_)_2_ for a study of the solid solubility at mole fractions *x*(PbMoO_4_) = 0.03 (left) and *x*(PbMoO_4_) = 0.97 (right).

During the cycling (*vide supra*) at *x* = 0.03 (Figure 12), it was found that the temperature of the polymorphic transition of Cs_2_MoO_4_ dropped from 833 ± 10 to 819 ± 10 K, which is in line with the temperature reported by Belyaev and Chikova^21^ for the eutectoid temperature belonging to β′ (819 K). The extent of solubility found here is lower than 3%, which is also in line with ref (21). At the eutectic temperature, no change in the temperature was found, contradicting the reported 13% solubility reported in the phase diagram in ref (21).

For the solid solubility at *x* = 0.97, no significant drop in the temperature was found in the present experiments (Figure 12). The analyzed onset temperatures decrease slightly but within the experimental error. The solubility of 5% Cs_2_MoO_4_ in PbMoO_4_ claimed by ref (21) is thus not substantiated by the present data.

#### Phase Diagram Equilibria in the Cs_2_MoO_4_–PbMoO_4_ Section

**Figure 13:**
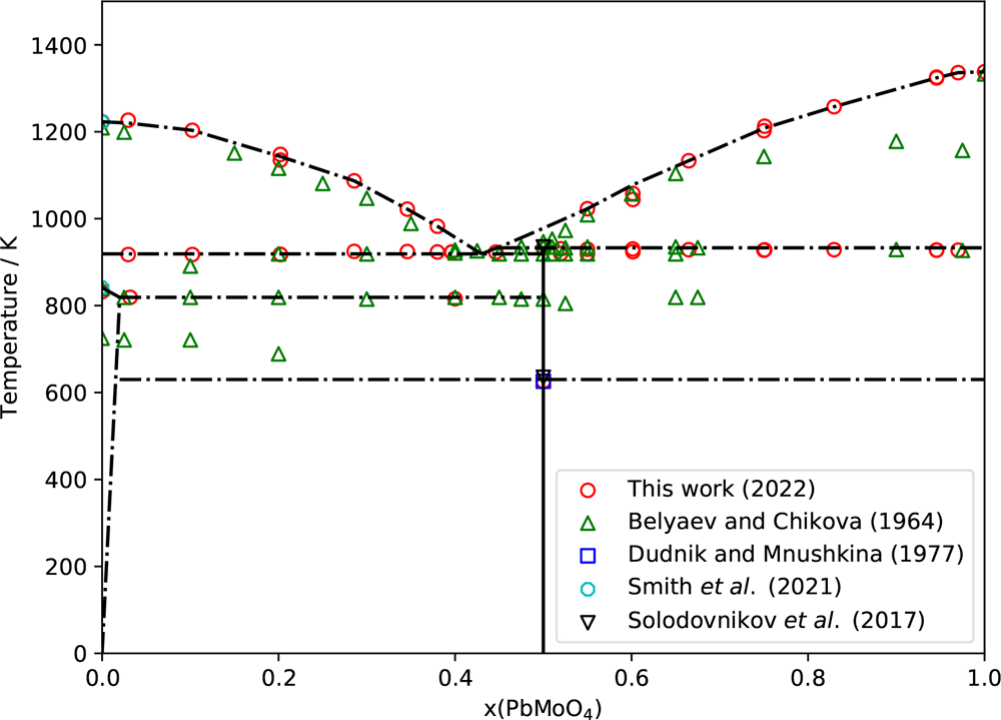
Sketch of the Cs_2_MoO_4_–PbMoO_4_ phase diagram as measured experimentally and compared with the literature.^3,7,21,37^ The lines drawn here depict the suggested phase equilibria.

The collected data in the pseudobinary section are listed in Table 7 with the associated invariant reactions. Based on the latter, a sketch of the phase diagram is proposed in Figure 13.

**Table 7:** Equilibrium Data in the Cs_2_MoO_4_–PbMoO_4_ System as Measured in This Work by DSC^a^

*x*(PbMoO_4_)	*T*/K	equilibrium	equilibrium reaction
0.000	837	polymorphism	α-Cs_2_MoO_4_ = β-Cs_2_MoO_4_
0.000	1222	congruent melting	β-Cs_2_MoO_4_ = L
0.030	819	eutectoid	Sol. sol. = β-Cs_2_MoO_4_ + Cs_2_Pb(MoO_4_)_2_
0.030	918	eutectic	β-Cs_2_MoO_4_ + Cs_2_Pb(MoO_4_)_2_ = L′
0.030	1227	liquidus	β-Cs_2_MoO_4_ + L′ = L
0.102	918	eutectic	β-Cs_2_MoO_4_ + Cs_2_Pb(MoO_4_)_2_ = L′
0.102	1203	liquidus	β-Cs_2_MoO_4_ + L′ = L
0.202	918	eutectic	β-Cs_2_MoO_4_ + Cs_2_Pb(MoO_4_)_2_ = L′
0.202	1141	liquidus	β-Cs_2_MoO_4_ + L′ = L
0.286	925	eutectic	β-Cs_2_MoO_4_ + Cs_2_Pb(MoO_4_)_2_ = L′
0.286	1087	liquidus	β-Cs_2_MoO_4_ + L′ = L
0.346	925	eutectic	β-Cs_2_MoO_4_ + Cs_2_Pb(MoO_4_)_2_ = L′
0.346	1022	liquidus	β-Cs_2_MoO_4_ + L′ = L
0.380	923	eutectic	β-Cs_2_MoO_4_ + Cs_2_Pb(MoO_4_)_2_ = L′
0.380	983	liquidus	β-Cs_2_MoO_4_ + L′ = L
0.396	923	eutectic	β-Cs_2_MoO_4_ + Cs_2_Pb(MoO_4_)_2_ = L′
0.400	816	eutectoid	Sol. sol. = β-Cs_2_MoO_4_ + Cs_2_Pb(MoO_4_)_2_
0.446	923	eutectic	β-Cs_2_MoO_4_ + Cs_2_Pb(MoO_4_)_2_ = L′
0.500	933	peritectic decomposition	Cs_2_Pb(MoO_4_)_2_= PbMoO_4_ + L″
0.520	930	peritectic	Cs_2_Pb(MoO_4_)_2_ = PbMoO_4_ + L″
0.550	930	peritectic	Cs_2_Pb(MoO_4_)_2_ = PbMoO_4_ + L″
0.550	1023	liquidus	PbMoO_4_ + L″ = L
0.601	930	peritectic	Cs_2_Pb(MoO_4_)_2_ = PbMoO_4_ + L″
0.601	1051	liquidus	PbMoO_4_ + L″ = L
0.665	929	peritectic	Cs_2_Pb(MoO_4_)_2_ = PbMoO_4_ + L″
0.665	1133	liquidus	PbMoO_4_ + L″ = L
0.750	928	peritectic	Cs_2_Pb(MoO_4_)_2_ = PbMoO_4_ + L″
0.750	1208	liquidus	PbMoO_4_ + L″ = L
0.830	929	peritectic	Cs_2_Pb(MoO_4_)_2_ = PbMoO_4_ + L″
0.830	1258	liquidus	PbMoO_4_ + L″ = L
0.950	928	peritectic	Cs_2_Pb(MoO_4_)_2_ = PbMoO_4_ + L″
0.950	1324	liquidus	PbMoO_4_ + L″ = L
0.970	928	peritectic	Cs_2_Pb(MoO_4_)_2_ = PbMoO_4_ + L″
0.970	1336	liquidus	PbMoO_4_ + L″ = L
1.000	1338	congruent melting	PbMoO_4_ = L

a Standard uncertainties on the composition are *u*[*x*(PbMoO_4_)] = 0.005; standard uncertainties on the temperature *T* are 5 K for pure compounds and 10 K for mixtures. The compositions with 0 < *x* < 0.5 and 0.5 < *x* < 1.0 are prepared by mixing Cs_2_Pb(MoO_4_)_2_ with respectively Cs_2_MoO_4_ and PbMoO_4_. No distinction is made between the room- and high-temperature phases of Cs_2_Pb(MoO_4_)_2_. Sol. sol. = solid solution at the Cs_2_MoO_4_-rich side.

The shape of the liquidus is in fair agreement with the data by Belyaev and Chikova, although their results tend to deviate toward the melting point of PbMoO_4_. Because, in the present results, a regular liquidus line near pure PbMoO_4_ is found, they are considered to be superior to the older results. Moreover, the melting points of the pure end members as measured in this work agree with the literature data.

The eutectic temperature reported here is 919 ± 10 K, in agreement with that in ref (21). The eutectic composition is proposed to be 0.40 < *x* < 0.45, aligning with *x* = 0.41, as proposed by Belyaev and Chikova.^21^

The peritectic temperature is 933 ± 5 K, within the error the same as that in ref (21). The second-order phase transition in Cs_2_Pb(MoO_4_)_2_ is drawn at 630 K. As can be seen from the absence of data points, the behavior of this transition close to Cs_2_MoO_4_ is still unknown.

Because the liquidus data were collected on the first heating cycle because of decomposition of Cs_2_Pb(MoO_4_)_2_, data for the eutectoid line was collected in separate multiple cycle measurements; the temperature found matches with that in ref (21).

## Conclusions

The phase diagram Cs_2_MoO_4_–PbMoO_4_ and the compound Cs_2_Pb(MoO_4_)_2_ were subjected to (renewed) research. ND and XRD gave insight into the behavior of the cell parameters at room temperature and above, up to 773 K. The crystal structure of β-Cs_2_Pb(MoO_4_)_2_, the high-temperature phase, was elucidated. β-Cs_2_Pb(MoO_4_)_2_ was found to crystallize in the palmierite space group (No. 166). For the first time, the thermal expansion of Cs_2_Pb(MoO_4_)_2_ was measured. The thermal expansion parameters were found to be larger than the thermal expansion parameters of the fuel pin but smaller than those of the JOG phase. The XANES spectrum was measured around the Mo K-edge. It was found that the oxidation state of Mo is 6+. The phase diagram section Cs_2_MoO_4_–PbMoO_4_ was investigated using a combination of XRD and TG–DSC. The existence of other compounds besides Cs_2_Pb(MoO_4_)_2_ in the investigated temperature–composition–pressure window was excluded. The phase diagram was found to be qualitatively similar to the phase diagram by Belyaev and Chikova,^21^ but several features were changed or refined. The liquidus line was improved toward the melting of PbMoO_4_. The compound was found to decompose peritectically but not at the previously suggested mole fraction. Furthermore, the solid solubility near the end members was investigated. It was found that the extent of solid solubility at the Cs_2_MoO_4_ side as reported earlier was exaggerated and at maximum 3%. The solid solubility at the PbMoO_4_ side was excluded.

The obtained results enable us to draw a few conclusions with regard to scenario analysis of the clad breach in a LFR. First, with operating temperatures up to almost 900 K, the Pb–Cs–Mo–O chemistry takes place in the solid state; no volatile interaction products are expected. Second, the mechanical interaction due to thermal expansion of the quaternary compound will be moderate compared to the JOG-phase compound Cs_2_MoO_4_. Third, the first interaction chemistry will probably form a small solid solution of Pb in Cs_2_MoO_4_, after which the stoichiometric ratios of the elements present at the outer rim of the fuel pin and the oxygen potential will determine the reaction products. If the herein-obtained results will be combined with a description of the Gibbs energy of all phases present in the system, the obtained insights can serve as reference data for the thermodynamic assessment of the clad breach scenario for future LFRs.
